# Biological Detoxification of Mycotoxins by Lactic Acid Bacteria: Safeguarding Food from Fungal Contaminants

**DOI:** 10.3390/toxins18050236

**Published:** 2026-05-20

**Authors:** Nazia Tabassum, Minji Kim, Tae-Hee Kim, Du-Min Jo, Won-Kyo Jung, Young-Mog Kim, Fazlurrahman Khan

**Affiliations:** 1 Research Center for Marine Integrated Bionics Technology, Pukyong National University, Busan 48513, Republic of Korea; nazia99@pukyong.ac.kr (N.T.); minjikim@pknu.ac.kr (M.K.); taehee@pknu.ac.kr (T.-H.K.); wkjung@pknu.ac.kr (W.-K.J.); 2 Marine Integrated Biomedical Technology Center, The National Key Research Institutes in Universities, Pukyong National University, Busan 48513, Republic of Korea; 3 Ocean and Fisheries Development International Cooperation Institute, Pukyong National University, Busan 48513, Republic of Korea; 4 National Marine Biodiversity Institute of Korea (MABIK), Seochun 33662, Republic of Korea; dmjo@mabik.re.kr; 5 Major of Biomedical Engineering, Division of Smart Healthcare, College of Information Technology and Convergence and New-Senior Healthcare Innovation Center (BK21 Plus), Pukyong National University, Busan 48513, Republic of Korea; 6 Department of Food Science and Technology, Pukyong National University, Busan 48513, Republic of Korea; 7 International Graduate Program of Fisheries Science, Pukyong National University, Busan 48513, Republic of Korea; 8 Interdisciplinary Program of Marine and Fisheries Sciences and Convergent Technology, Pukyong National University, Busan 48513, Republic of Korea

**Keywords:** mycotoxins, lactic acid bacteria, biological detoxification, food safety, cell wall adsorption, biotransformation

## Abstract

Mycotoxins are one of the biggest threats to global food safety, public health, and economic stability. More than 400 mycotoxins have been found to be secondary metabolites of toxigenic fungi, mostly from the genera *Aspergillus*, *Fusarium*, *Penicillium*, and *Alternaria*. Aflatoxins (AFs), ochratoxin A (OTA), deoxynivalenol (DON), zearalenone (ZEA), fumonisins (FBs), patulin (PAT), and T-2/HT-2 toxins are the most dangerous to the health of people and animals. Conventional physical and chemical decontamination methods are only partially effective and can reduce food quality, leave toxic residues, or be too expensive for smallholder food systems. Recent studies have shown that the application of lactic acid bacteria (LAB) as a biological detoxification method is a safe, cost-effective, and environmentally friendly option, and has a long history of safe use in fermented foods. Selected strains or taxonomic units have been granted GRAS status by the FDA or QPS (Qualified Presumption of Safety) status by EFSA. However, their use for mycotoxin detoxification still requires strain-level safety assessment and efficacy validation in the intended food matrix. There are several mechanisms by which LAB employ to reduce the bioavailability of mycotoxins in food systems: (i) physical adsorption via cell wall components such as peptidoglycan, teichoic acids, and exopolysaccharides; (ii) enzymatic biotransformation that may produce non-toxic or less-toxic metabolites, though the safety of degradation products requires case-by-case toxicological assessment; (iii) antifungal metabolite production that inhibits fungal growth and mycotoxin biosynthesis; and (iv) competitive exclusion of toxigenic fungi during fermentation. This comprehensive review examines the existing evidence on the detoxification of major food mycotoxins by LAB, with an emphasis on mechanisms, strain-specific efficacy, food-matrix applications, and factors that affect detoxification efficacy. Discussion has also been made of translating in vitro findings to in vivo settings and food-scale applications, alongside regulatory frameworks, current challenges, and future research directions. The review also suggests ways to combine LAB with new technologies, such as encapsulation, genetic engineering, and fermentation optimization, to make food systems safer by synergistically controlling mycotoxins.

## 1. Introduction

Mycotoxins are toxic secondary metabolites produced by filamentous fungi during the colonization of food crops, commodities, and animal feeds. The term ‘mycotoxin’ was first coined in 1962 following the ‘Turkey X disease’ outbreak in the United Kingdom that killed approximately 100,000 turkeys and was traced to aflatoxin-contaminated Brazilian peanut meal [1]. Since then, more than 400 structurally diverse mycotoxins have been characterized, with new analogs continuing to be identified [2]. Mycotoxin contamination represents a significant global challenge with substantial economic and health implications. The widely cited FAO estimate of 25% global food crop contamination appears valid when considering regulatory limits, though actual detectable contamination may reach 60–80% [3]. Economic impacts are severe, with annual costs estimated at USD 100 million globally, and regions accounting for 70% of global nut and dried fruit imports face particular challenges due to cross-border trade restrictions [4]. Mycotoxins pose serious health risks, including carcinogenicity, nephrotoxicity, and hepatotoxicity [5]. Developing countries like Ethiopia face disproportionate impacts due to inadequate control systems [6].

Aflatoxin exposure through contaminated food represents a significant global health concern, particularly in sub-Saharan Africa and Southeast Asia, where chronic dietary exposure to AFB1, often combined with hepatitis B infection, substantially increases hepatocellular carcinoma (HCC) risk [7]. Quantitative risk assessments demonstrate varying regional burdens, with studies in Nigeria estimating 1.77–2.8 HCC cases per 100,000 population annually from aflatoxin exposure through staple foods like maize and groundnuts [8]. In China, dietary aflatoxin exposure contributes 0.125–0.4 extra HCC cases per 100,000 persons yearly [9,10]. Aflatoxin-associated HCCs exhibit distinct genetic features, including specific mutations and increased PD-L1 expression [11].

The primary toxigenic fungi affecting food commodities include *Aspergillus flavus*, *A. parasiticus*, *A. ochraceus*, *Fusarium graminearum*, *Fusarium verticillioides* (formerly *F. moniliforme*), *Penicillium verrucosum*, *P. expansum*, and various *Alternaria* species [12]. Environmental factors, including temperature, relative humidity, water activity, substrate composition, and post-harvest handling practices, strongly modulate fungal growth and mycotoxin production [13,14]. Climate change is projected to exacerbate mycotoxin contamination by expanding the geographic range of toxigenic fungi and altering crop vulnerability [15]. Cereals (maize, wheat, barley, sorghum), nuts (peanuts, tree nuts), spices, dried fruits, cocoa, coffee, and dairy products (via feed-to-food carry-over of aflatoxin M1) are the most commonly affected commodities [16,17].

Current regulatory limits for mycotoxins in food have been established by agencies including the European Commission (EC), the US Food and Drug Administration (FDA), Codex Alimentarius, and numerous national food safety authorities. Despite stringent regulations, mycotoxin incidents continue to occur globally due to inadequate pre-harvest control, poor post-harvest storage infrastructure, and the inherent stability of many mycotoxins to conventional food processing [18]. Physical methods such as sorting, washing, heat treatment, and UV irradiation achieve limited success, while chemical decontamination using ammonia, ozone, or chlorinating agents raises concerns about residue safety, nutrient loss, and organoleptic quality [19,20].

Biological detoxification, the use of living microorganisms or their metabolites to degrade, bind, or sequester mycotoxins, has attracted considerable research attention as a safer, selective, and eco-friendly alternative [21,22]. Among the microbial agents evaluated, lactic acid bacteria (LAB) stand out for their long history of safe use in food fermentation, GRAS/QPS (Qualified Presumption of Safety) status, probiotic properties, and multifaceted detoxification capabilities [23]. LAB colonize diverse ecological niches—fermented foods, dairy products, plant surfaces, intestinal environments—and collectively encompass species of *Lactiplantibacillus plantarum* (formerly *Lactobacillus plantarum*), *Lactobacillus acidophilus*, *Lacticaseibacillus casei* (formerly *Lactobacillus casei*), *Lactobacillus fermentum*, *Lacticaseibacillus rhamnosus* (formerly *Lactobacillus rhamnosus*), *Limosilactobacillus reuteri*, *Lactococcus lactis*, *Streptococcus thermophilus*, *Leuconostoc mesenteroides*, *Enterococcus faecium*, *Pediococcus acidilactici*, and many others [24].

LAB contribute to food safety through a variety of mechanisms, including lactic acid production (lowering pH), secretion of bacteriocins and hydrogen peroxide, competition for nutrients, and direct mycotoxin detoxification via adsorption and enzymatic biotransformation [25,26,27]. Several decades of research, from pioneering in vitro binding studies in the 1990s and 2000s to recent in vivo and food-matrix studies, have established a solid evidence base for the role of LAB in reducing mycotoxin bioavailability. Despite this progress, key challenges remain: variability in strain-level detoxification capacity, insufficient in vivo validation, limited knowledge of degradation product safety, and a lack of standardized protocols for industrial application [28,29].

This review provides a comprehensive, critically assessed overview of current knowledge on LAB-mediated mycotoxin detoxification. It covers the major mycotoxins of food safety concern, the taxonomy and safety status of detoxification-competent LAB strains, the mechanistic basis of detoxification, mycotoxin-specific evidence, food application contexts, regulatory frameworks, and an outlook on emerging integrated strategies. This review also compiles the toxicity profiles of major LAB-mediated mycotoxin degradation products, an aspect largely absent from existing reviews, to assess whether reported detoxification translates into genuine risk reduction. The goal is to serve as a reference for food scientists, microbiologists, regulatory scientists, and food industry professionals seeking to harness LAB to enhance food safety.

## 2. Major Mycotoxins in Food: Occurrence, Chemistry, and Health Impacts

Mycotoxins of regulatory significance in human food can be grouped into several chemical classes according to their biosynthetic origins and structural features. Table 1 summarizes the major mycotoxins, their fungal producers, affected food commodities, principal health effects, and current EU maximum limits, which serve as representative regulatory standards.

Aflatoxin B1 (AFB1) is widely recognized as the most potent naturally occurring carcinogen, classified as a Group 1 carcinogen by the IARC, with significant genotoxic and hepatocarcinogenic effects in humans and animals [57,58]. AFB1 contamination is a global concern, affecting up to 25% of crops, particularly in regions such as Africa and Asia, and is exacerbated by climate change [59,60]. AFB1 undergoes hepatic metabolic activation by cytochrome P450 enzymes (CYP1A2, CYP3A4) to form the reactive AFB1-8,9-exo-epoxide, which binds covalently to guanine residues in DNA, generating AFB1-N7-guanine adducts, driving G→T transversions and inducing characteristic inactivating mutations in the TP53 tumor suppressor gene [30]. OTA is a chlorinated dihydroisocoumarin linked to phenylalanine; its nephrotoxic and immunosuppressive properties are well-established, and chronic dietary exposure is associated with urothelial and kidney tumors in rodent models [35]. DON, also called vomitoxin, is a type B trichothecene that inhibits eukaryotic ribosomal peptidyltransferase activity by interacting with the 60S ribosomal subunit, triggering a ribotoxic stress response, cytokine induction, and intestinal epithelial barrier dysfunction [38,39]. ZEA is a macrocyclic β-resorcyclic acid lactone that binds estrogen receptors (ERα, ERβ) and interferes with the hypothalamic-pituitary-gonadal axis, causing reproductive failure, hyperestrogenism, and infertility, particularly in swine [43]. Fumonisins, particularly FB1, structurally mimic sphingoid bases (sphinganine and sphingosine) and competitively inhibit ceramide synthase, thereby disrupting sphingolipid metabolism, which is critical for membrane structure and signal transduction [45]. Patulin (PAT), predominantly found in apple products, is an alkylating compound that reacts with protein thiol groups, induces oxidative stress, and suppresses immune function [47,48]. T-2 toxin is a type A trichothecene mycotoxin produced by various Fusarium species and is considered the most toxic member of the trichothecene family [61,62]. Emerging mycotoxins, including enniatins, beauvericin, alternariol, and others, are increasingly detected in European cereal monitoring data [54,55].

## 3. Lactic Acid Bacteria: Classification, Ecology, and Safety Status

Lactic acid bacteria constitute a phylogenetically and ecologically diverse group of Gram-positive, non-spore-forming, catalase-negative, facultatively anaerobic bacteria united by their characteristic production of lactic acid as the primary or sole end-product of carbohydrate fermentation [63]. The taxonomy of LAB has undergone substantial reclassification through comparative 16S rRNA gene analysis and whole-genome sequencing; historically recognized genera such as *Lactobacillus* have been reorganized into 23 novel genera, and the original genus *Lactobacillus* was retained, including *Lactiplantibacillus*, *Lacticaseibacillus*, *Ligilactobacillus*, *Limosilactobacillus*, *Lentilactobacillus*, and others [64]. Major genera include *Lactobacillus sensu* stricto, *Lactiplantibacillus*, *Lacticaseibacillus*, *Limosilactobacillus*, *Leuconostoc*, *Pediococcus*, *Lactococcus*, *Streptococcus* (thermophilic species), *Enterococcus*, *Oenococcus*, and *Weissella* [63,65]. LAB are ubiquitous in fermented foods (yogurt, kefir, cheese, sourdough, sauerkraut, kimchi, fermented meats, wine, pickles), plant materials, soil, and the gastrointestinal tracts of humans and animals [66]. Many LAB strains have a long history of safe use in food fermentation, and selected strains have been accorded GRAS status by the FDA (USA) or Qualified Presumption of Safety (QPS) by EFSA (EU) [67]. The European Food Safety Authority periodically publishes updated lists of QPS-recommended microorganisms; LAB genera, including *Lactiplantibacillus*, *Lacticaseibacillus*, *Limosilactobacillus*, *Lactococcus*, *Leuconostoc*, *Pediococcus*, *Streptococcus thermophilus*, and *Bifidobacterium*, are included under QPS status [68].

Beyond acidification, LAB produce a diverse array of antimicrobial compounds, including bacteriocins (nisin, plantaricin, pediocin), hydrogen peroxide, diacetyl, reuterin, and short-chain fatty acids, which collectively underpin their biopreservative action in food [27,69,70,71]. Several LAB strains have been approved as probiotic supplements and functional food ingredients, demonstrating the robust clinical evidence base supporting their safety in human consumption [72,73]. This safety profile critically differentiates LAB-based detoxification from other biological control agents and supports the translation of well-characterized strains to food-scale applications, though use for mycotoxin detoxification still requires strain-specific safety assessment, efficacy validation, and regulatory approval for the intended food matrix and application. Table 2 provides a consolidated summary of the key LAB species employed in mycotoxin detoxification research, integrating taxonomic, safety, food application, detoxification efficacy, mechanistic, and bibliographic information in a single reference.

## 4. Mechanisms of Mycotoxin Detoxification by Lactic Acid Bacteria

### 4.1. Physical Adsorption and Cell Wall Binding

**Figure 1:**
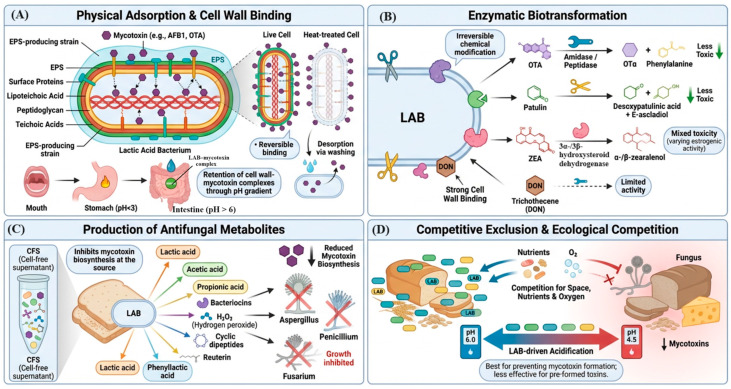
Fungal mycotoxin detoxification by several species of lactic acid bacteria via several mechanisms, such as (**A**) physical adsorption and cell wall binding, (**B**) enzymatic biotransformation, (**C**) production of antifungal metabolites, and (**D**) competitive exclusion and ecological competition.

Physical adsorption, the non-covalent binding of mycotoxin molecules to bacterial surface components without chemical transformation (Figure 1A), is the most extensively documented mechanism of LAB-mediated mycotoxin detoxification, particularly for aflatoxins [74,82]. The bacterial cell wall of LAB, which lacks an outer membrane, unlike Gram-negative bacteria, is composed predominantly of peptidoglycan, interspersed with teichoic acids, lipoteichoic acids, surface proteins, and exopolysaccharides (EPS) that collectively provide functional groups (hydroxyl, amino, carboxyl, phosphate) capable of interacting with mycotoxins. Early work by El-Nezami et al. [74] demonstrated that *L. rhamnosus* GG and *L. rhamnosus* LC-705 could remove 80–90% of AFB1 from aqueous solutions through physical binding, with binding occurring rapidly (within minutes) and being largely extracellular and surface-mediated [74].

The specific cell wall components mediating adsorption have been characterized through comparative binding studies using isolated fractions (whole cells, cell wall preparations, peptidoglycan, EPS, teichoic acids). Haskard et al. [77] using competitive inhibition ELISA demonstrated that AFB1 binding is surface-mediated and accessible to an anti-AFB1 antibody, pointing to extracellular and reversible complex formation [77]. Studies with *L. plantarum* T3 identified peptidoglycan as the primary binding site, with adsorption rates of 84% for heat-inactivated cells, 87.8% for isolated peptidoglycan, and 77% for crude cell wall preparations at an AFB1 concentration of 1 µg/mL [26]. The carbohydrate moieties within peptidoglycan (*N*-acetylmuramic acid and *N*-acetylglucosamine), as well as the attached teichoic acid chains, are proposed to interact with the coumarin ring of aflatoxin via hydrogen bonding and hydrophobic interactions [77,79,89].

Exopolysaccharide production by certain LAB strains has been identified as an additional binding mechanism. EPS-producing strains demonstrate significantly higher mycotoxin removal rates than non-EPS-producing strains for AFB1 and OTA under comparable conditions [33]. Heat or acid treatment of bacteria (producing ‘non-viable’ forms) frequently enhances or maintains binding capacity compared to viable cells, since cellular disruption exposes additional internal binding sites and eliminates potential metabolic liberation of bound toxin [79]. This observation is important for practical food applications, where heat-killed LAB preparations may be more stable and suitable for incorporation into processed foods. The binding of mycotoxins to LAB cell walls is generally considered reversible, as repeated washing with aqueous solutions leads to incremental desorption. However, the stability of complexes varies substantially by strain, mycotoxin structure, and environmental conditions (pH, ionic strength, temperature), and some complexes exhibit sufficient stability to persist through simulated gastrointestinal conditions, supporting the proposal that LAB-mycotoxin complexes could reduce absorption in the gut even when ingested after food processing [36,80]. Studies simulating gastrointestinal transit (using sequential pH stages representing salivary, gastric, and intestinal conditions) suggest that AFB1 bound to *L. rhamnosus* RC007 is maximally retained under gastric and intestinal pH conditions [93].

### 4.2. Enzymatic Biotransformation

Enzymatic biotransformation has emerged as the most promising detoxification approach due to its specificity, environmental friendliness, and reusability, compared with physical and chemical methods (Figure 1B) [94,95]. LAB encode diverse metabolic enzymes, including esterases, lactonases, oxidoreductases, peroxidases, and hydrolases that can catalyze reactions such as hydrolysis, oxidation, reduction, de-epoxidation, methylation, and decarboxylation of functional groups in mycotoxins [88,96,97].

Ochratoxin A (OTA) degradation by LAB and other microorganisms represents a promising biodetoxification approach for contaminated food and feed products. Multiple bacterial strains produce amidohydrolases that cleave OTA’s amide bond, converting it to the less toxic ochratoxin α (OTα) and L-β-phenylalanine [98].

Patulin degradation by LAB is an important food-safety application, given patulin’s prevalence in apple-based products. *Lactobacillus* species ferment apple juice during patulin reduction not only by adsorption but through enzymatic attack on the α,β-unsaturated lactone ring of patulin, producing the less toxic desoxypatulinic acid and E-ascladiol as primary degradation products [99]. Research has demonstrated significant patulin degradation capabilities in apple juice using various biological approaches. Lactic acid bacteria strains show particularly high efficacy, with *L. casei* YZU01 achieving complete degradation of 10 μg/mL patulin in raw apple juice after 36 h [100], and inactivated *L. kefiranofacien* achieving 93% removal at 100 μg/L concentration [101].

Research on zearalenone (ZEA) detoxification by lactic acid bacteria reveals two distinct enzymatic pathways for mycotoxin removal. Studies demonstrate that LAB strains employ both adsorption and biodegradation mechanisms [102,103,104]. The adsorption pathway involves hydrophobic interactions between ZEA and bacterial cell wall components, including exopolysaccharides, proteins, and lipids, with electrostatic forces playing minimal roles [103,105]. The biodegradation pathway involves enzymatic transformation, in which LAB convert ZEA into metabolites such as α-ZOL and β-ZOL via esterase activity [102,105]. *Lactobacillus plantarum*, *L. paracasei*, and *L. buchneri* strains show particularly high ZEA removal efficiency (68–78%) under optimal conditions [102,105].

Biotransformation of trichothecenes (DON, T-2 toxin) is particularly challenging due to the stability of the trichothecene ring system under physiological conditions. Some LAB strains demonstrate limited DON transformation via de-epoxidation (reduction of the 12,13-epoxy group to form de-epoxy DON, DOM-1) catalyzed by specialized anaerobic consortia, but the intrinsic de-epoxidase activity of well-characterized LAB is limited; their primary role is adsorption or reduction of DON production by inhibiting Fusarium growth [106]. Cell wall adsorption dominates over enzymatic degradation for DON in LAB systems, although combinatorial approaches involving LAB co-inoculated with DON-degrading bacteria (e.g., *Devosia riboflavina*) have shown synergistic detoxification [40].

### 4.3. Production of Antifungal Metabolites

An important indirect mechanism of mycotoxin reduction by LAB is the inhibition of toxigenic fungal growth through the production of antifungal metabolites, thereby preventing mycotoxin biosynthesis at the source (Figure 1C) [107]. This mechanism is distinct from the direct detoxification of pre-formed mycotoxins, but is arguably more impactful in the context of food fermentation and biopreservation, where LAB colonization precedes or coincides with fungal contamination events.

Organic acids (lactic acid, acetic acid, propionic acid, phenyllactic acid) produced by LAB lower the pH of food matrices, inhibiting mycotoxigenic fungi, including *Aspergillus*, *Penicillium*, and *Fusarium* species at pH values below 4.5–5.0 [108]. Phenyllactic acid (PLA) produced by *L. plantarum* strains demonstrates significant antifungal activity against various food spoilage fungi. Multiple studies confirm PLA’s broad-spectrum antimicrobial properties, effectively inhibiting *Aspergillus flavus*, *A. parasiticus*, *Penicillium roqueforti*, and other pathogenic molds [109,110,111]. PLA shows particularly strong effects against aflatoxin B1 (AFB1) production, with inhibition rates reaching 89–91% [111,112]. The mechanism involves dose-dependent disruption of fungal cell membrane integrity and downregulation of AFB1 biosynthesis genes [113]. *L. plantarum* strains producing PLA have been successfully applied as biocontrol agents in cereal-based products, including bread and oat beverages, demonstrating practical food preservation applications [109,114].

Bacteriocins produced by LAB (nisin from *L. lactis*, plantaricin from *L. plantarum*, pediocin from *Pediococcus acidilactici*) are ribosomally synthesized antimicrobial peptides primarily active against Gram-positive bacteria, but several have also demonstrated antifungal activity affecting hyphal growth and conidiophore development [115]. Hydrogen peroxide generated by LAB under aerobic conditions denatures fungal enzymes involved in mycotoxin biosynthesis, thereby inhibiting aflatoxin accumulation in model grain substrates [116]. The antifungal mechanisms involve production of organic acids, including lactic acid, phenyllactic acid, and hydroxyphenyllactic acid, which effectively suppress AFB1 production by 73.7–99.7% [111,114,117]. Cyclic dipeptides cyclo(l-Phe-l-Pro) and cyclo(l-Phe-trans-4-OH-l-Pro) produced by *L. plantarum* MiLAB 393 isolated from grass silage have been reported to inhibit *A. fumigatus* growth [118]. Reuterin (3-hydroxypropionaldehyde), produced by *Limosilactobacillus reuteri* through glycerol metabolism, demonstrates broad-spectrum antifungal activity against diverse microorganisms. Studies show reuterin inhibits yeasts and molds at concentrations ≤ 11 mM, with fungicidal activity at ≤15.6 mM [119]. The compound effectively inhibits various fungal species, including *Fusarium oxysporum*, *Colletotrichum gloeosporioides*, *Alternaria alternata*, and *Penicillium digitatum* [120]. The cell-free supernatants (CFS) of many LAB strains, which contain a complex mixture of secreted metabolites, exhibit antifungal activity that often exceeds that of individual pure compounds, suggesting synergistic interactions among lactic acid, acetic acid, PLA, proteinaceous antifungal factors, and other secreted compounds [121].

### 4.4. Competitive Exclusion and Ecological Competition

In fermented food systems, LAB exert mycotoxin-reducing effects through competitive exclusion of toxigenic fungi, outcompeting fungi for substrate nutrients, oxygen, and colonization niches before they can proliferate and produce mycotoxins (Figure 1D) [118]. Inoculating cereals, fruits, or dairy substrates with active LAB starter cultures shifts the microbial ecology of the substrate toward bacterial dominance, reducing the initial fungal load and impeding further fungal growth during fermentation [85]. The efficacy of this mechanism depends on the relative growth rates of LAB versus toxigenic fungi under substrate conditions, as well as the timing of LAB inoculation relative to fungal contamination events. In sourdough fermentation, the rapid acidification mediated by LAB (pH dropping from 6.0 to below 4.5 within hours) creates conditions inhospitable to *Penicillium* and *Aspergillus* growth, significantly reducing aflatoxin contamination compared to non-fermented controls [86,122]. Similar competitive effects have been documented in fermented maize (African ogi, kenkey, akamu), fermented cassava, silage, and fermented beverages [84]. The competitive exclusion mechanism, however, is limited in addressing mycotoxin contamination already present prior to fermentation, underscoring the importance of combining LAB-based strategies with pre-fermentation grain sorting and decontamination practices [123].

## 5. LAB-Mediated Detoxification of Specific Mycotoxins: Evidence and Efficacy

### 5.1. Aflatoxins

The aflatoxins, particularly AFB1, AFB2, AFG1, AFG2, and the metabolic derivative AFM1, have been the most extensively studied group of mycotoxins in research on LAB detoxification. Seminal work by El-Nezami et al. [74,124] established that *L. rhamnosus* GG and *L. rhamnosus* LC-705 could remove >80% of AFB1 from liquid media through surface binding, and that the bound AFB1-bacteria complex was stable through simulated gastrointestinal conditions, reducing AFB1 genotoxicity in cell-based assays [74,124]. Gratz et al. [125] demonstrated in an ex vivo porcine intestinal model that a mixture of *L. rhamnosus* GG and *Propionibacterium freudenreichii* ssp. *shermanii* JS reduced AFB1 absorption across the intestinal wall, supporting the in vivo relevance of in vitro binding assays [126].

A critical development in AFB1 binding research has been the systematic screening of large LAB collections to identify high-performance strains [79]. Research demonstrates that *L. plantarum* strains effectively mitigate AFB1 toxicity through multiple mechanisms. *L. plantarum* T3 removed up to 68.5% of AFB1 at 1 µg/mL in vitro, with peptidoglycan identified as the principal binding site, achieving 87.8% removal [26]. Similarly, *L. plantarum* C88 exhibited strong AFB1-binding capacity and increased fecal AFB1 excretion in mice [127]. In vivo studies confirm hepatoprotective effects, with oral administration significantly normalizing serum liver enzymes (ALT, AST), reducing oxidative stress markers (MDA), and restoring gut microbiota balance [26,128]. Compared with unsupplemented controls, LAB increased GSH, GST, and GR by 11.6–86.1%; in AFB1-challenged birds, LAB supplementation restored these enzyme levels by 24.1–146.9% compared with the AFB1-only group [129]. Probiotic supplementation promotes gut microbial homeostasis in broiler chickens exposed to AFB1 [130] and reduces AFB1 residues in liver and kidneys while ameliorating histopathological changes [131]. The detoxification of AFM1 in dairy products, a significant public health concern given contamination in milk, cheese, and infant formula, has been demonstrated using *L. acidophilus*, *L. rhamnosus*, *Streptococcus thermophilus*, and *Bifidobacterium* species. Govaris et al. [87] showed *L. delbrueckii* subsp. *bulgaricus* and *S. thermophilus* in yogurt starter cultures reduced AFM1 by approximately 22–32% during fermentation at 42 °C. A key challenge in dairy applications is the reversibility of AFM1 binding during whey separation and washing steps in cheese manufacturing [87].

Co-culture and mixed-strain studies frequently demonstrate additive or synergistic AFB1 removal compared to single-strain applications [79]. Escrivá et al. [24] reported that *Lactobacillus* strains isolated from goat milk whey, when added as natural ingredients in bread dough, reduced AFB1 and OTA concentrations by 30–60% compared to controls without LAB, demonstrating direct applicability in baked goods production [24]. Sourdough fermentation using *L. plantarum* and *L. fermentum* starter cultures has been shown to reduce AFB1 in wheat, maize, and sorghum flours by 40–70%, with reductions correlating with fermentation time and acidification rate [132].

### 5.2. Ochratoxin A

OTA detoxification by LAB involves both adsorption and enzymatic hydrolysis, with the relative contributions depending on the strain and food matrix. Early studies by Piotrowska [133] showed that various *Lactobacillus* species could remove 16.9-35% of OTA from MRS broth via binding mechanisms, with no evidence of degradation products, suggesting that adsorption was responsible for the removal.

Wine is a major OTA-contaminated product, primarily produced by *Aspergillus carbonarius* in European vineyards [134,135]. The vinification process generally reduces OTA levels compared to grapes, though not all steps contribute equally to this reduction [134]. LAB shows promise for OTA biodetoxification through both degradation and adsorption mechanisms [98,136]. *Lactobacillus* strains can effectively carry out malolactic fermentation in wines with pH > 3.5 [137] and demonstrate significant OTA removal. *Lactobacillus rhamnosus* Bm01 removed 83.58% of OTA primarily through cell wall adsorption within 48 h [138], while actinobacterial strains achieved 67–83% OTA reduction by suppressing OTA gene expression [139]. The formation of OTα (ochratoxin alpha) as a degradation product deserves attention: while OTα is substantially less cytotoxic than OTA in mammalian cell lines, it retains some mutagenic activity and phytotoxicity, and its accumulation in food at high concentrations should be monitored [98].

### 5.3. Deoxynivalenol (DON)

DON detoxification by LAB is predominantly mediated by cell wall adsorption, as the trichothecene ring of DON is resistant to most conventional LAB metabolic enzymes [106]. Niderkorn et al. [40] showed that fermentative bacteria, including LAB (*L. fermentum*, *L. plantarum*, and *Enterococcus faecalis*), adsorbed DON, T-2 toxin, and ZEA in vitro with rates highly dependent on strain and toxin structure, suggesting cell wall moiety differences govern specificity [40]. Franco et al. [41] demonstrated that LAB strains from Brazilian fermented foods inhibited *F. graminearum* growth and DON production, with antifungal metabolites in cell-free supernatants contributing to reduced DON levels in artificially contaminated wheat [41]. In fermented sourdough systems, the combined effects of acidification and physical binding reduced DON contamination in naturally Fusarium-infected wheat by up to 40% [86]. Mischler et al. [55] systematically evaluated 238 LAB and *Bacillus* strains for their ability to reduce DON and the emerging mycotoxin enniatin B, finding that while DON removal by LAB was modest (10–30%), the combination of LAB cell wall adsorption and *Bacillus* enzymatic degradation achieved synergistic reductions [55].

### 5.4. Zearalenone (ZEA)

Lactic acid bacteria demonstrate significant potential for ZEA detoxification through both adsorption and biodegradation mechanisms. Multiple *Lactobacillus* species, including *L. plantarum*, *L. paracasei*, *L. acidophilus*, and *L. buchneri*, show ZEA removal efficiencies ranging from 23% to 91.7% [76,102,103,105]. Heat treatment generally enhances removal efficiency by modifying bacterial cell structure [76,103]. The primary mechanism involves hydrophobic interactions rather than electrostatic forces, with cell wall components including exopolysaccharides, proteins, and lipids participating in ZEA binding [103,105]. Some strains also biotransform ZEA into metabolites like α-zearalanol and zearalenone sulfate [76,105]. *L. plantarum* strains show particularly strong ZEA removal capabilities, with removal rates ranging from 65–90% depending on conditions and treatment methods [75,76,103]. The concern that ZEA reduction by carbonyl reductases produces α-zearalenol (more estrogenic) rather than the less estrogenic β-zearalenol necessitates careful metabolite profiling when evaluating LAB strains for ZEA detoxification [44]. Lactonase-mediated ring opening of ZEA to yield non-estrogenic linear products is a more desirable biotransformation pathway, and LAB strains producing relevant lactonase activities are under investigation [140]. The efficacy of ZEA removal in actual food fermentation (silage, sourdough, fermented maize) has been demonstrated in multiple studies, with average reductions of 30–70% under optimal fermentation conditions [141].

### 5.5. Fumonisins

Research demonstrates that fumonisin B1 and B2 (FB1 and FB2) detoxification by LAB shows moderate efficiency compared to other mycotoxins, with binding rates typically ranging from 10–40% due to limited hydrophobic interaction sites in the polyol backbone and tricarballylic acid side chains of fumonisins [142,143]. Enzymatic approaches using fumonisin esterases show greater promise, with novel carboxylesterases such as FumDSB achieving significant detoxification by hydrolyzing fumonisin B1 into less toxic metabolites [144]. Commercial fumonisin esterase FumD achieves ≥80% reduction in contaminated maize by de-esterifying tricarballylic acid units [145]. Structural studies reveal these enzymes exhibit regioselective cleavage and high catalytic efficiency with low K_M_ values (4.76–44.3 μM) suitable for environmental contamination levels [146]. While LAB binding remains strain-specific and moderately effective, enzymatic detoxification represents a more promising biotechnological approach for fumonisin control [78,147]. Studies show that LAB has significant potential to control *Fusarium verticillioides* growth and reduce fumonisin contamination in cereals through multiple mechanisms. LAB strains produce antifungal metabolites, including lactic acid and other bioactive compounds, that effectively inhibit *Fusarium* growth [78,148]. Studies show LAB can reduce mycotoxin production by 73.7–99.7% in corn substrates [114], with specific strains like *Lactiplantibacillus plantarum* and *Limosilactobacillus fermentum* demonstrating strong antagonistic activity against *F. verticillioides* on wheat and corn [149]. Machine learning models have been developed to predict fungal growth inhibition by LAB strains [150]. The biocontrol mechanisms include direct antifungal activity through cell-free supernatants and mycotoxin detoxification capabilities [151,152]. This biological approach offers a GRAS-status alternative to chemical preservatives for managing *F. verticillioides* and fumonisin contamination in cereal-based foods [153].

### 5.6. Patulin (PAT)

PAT reduction by LAB has been studied primarily in the context of apple juice and fermented apple products. Research on PAT contamination in apple products reveals variable occurrence rates and concentrations across different regions and analytical methods. Belgian surveillance of 103 apple juices found PAT in 54% of samples, with concentrations up to 191 µg/L and five samples exceeding regulatory limits [154]. Pakistani studies reported higher contamination rates, with 58.9% of apple samples containing PAT at a mean level of 49.8 µg/kg and 27.3% exceeding the 50 µg/kg limit [155]. In contrast, Taiwanese imported apple products showed lower contamination, with only 5.84% of apple juices containing detectable PAT, at a mean level of 1.7 µg/kg [156]. Brazilian samples showed even lower contamination rates, with PAT levels below the quantification limit in 24 analyzed apple juice samples [157]. Advanced analytical methods using LC-MS/MS have been successfully developed and validated for PAT detection, achieving detection limits as low as 0.5 µg/L [158,159].

Research demonstrates that *Lactobacillus* strains effectively reduce PAT contamination in apple-based systems through enzymatic degradation and adsorption. The unsaturated lactone ring of PAT reacts with thiol-containing metabolites produced by LAB (glutathione, cysteine, homocysteine), forming irreversible addition products with substantially reduced toxicity [49]. Wei et al. [160] showed that *L. plantarum* CCFM1287 reduced PAT concentrations by 85.09% in MRS medium, following first-order degradation kinetics. Zoghi et al. [161] reported that *L. acidophilus* and *L. plantarum* achieved 91.23% PAT removal from apple juice, with surface-layer proteins serving as binding sites for adsorption. The degradation process produces less toxic metabolites, including E-ascladiol and desoxypatulinic acid [162,163,164]. These transformation products demonstrate significantly reduced cytotoxicity compared to PAT [163,165]. Multiple studies confirm that enzymatic conversion via short-chain dehydrogenases/reductases transforms PAT to E-ascladiol [162,164], while maintaining apple juice quality during treatment [161,162,164].

### 5.7. T-2 Toxin and HT-2 Toxin

Type A trichothecenes (T-2 and HT-2 toxins) are highly toxic trichothecene mycotoxins produced by *Fusarium* species that contaminate cereal grains worldwide [62,166]. These toxins pose significant health risks, with T-2 being the most toxic trichothecene, causing immunotoxicity, neurotoxicity, and reproductive toxicity [62,167]. Human dietary exposure is highest among vulnerable populations, with toddlers and infants showing upper-bound estimates of 64.8 and 62.9 ng/kg body weight per day, respectively [166]. The highest concentrations occur in oats and oat-containing commodities, with cereal flakes and fine bakery products being major contributors to exposure [166]. Infants consuming maize-based complementary foods face particular risk, with exposure varying by geographical region [168]. Currently, mycotoxin occurrence in food crops indicates widespread contamination, with detectable levels in 60–80% of analyzed samples [3]. Effective decontamination strategies remain challenging, as complete elimination may not be possible [62]. Research demonstrates that LAB strains effectively adsorb various mycotoxins through cell wall interactions. *L. plantarum* T3 achieved 68.5% AFB1 removal, with peptidoglycan showing 87.8% adsorption capacity [26]. *L. acidophilus* and *L. rhamnosus* combinations demonstrated significant adsorption of AFB1, OTA, and ZEA, though stability decreased after solvent washing [169]. Kefir-derived *L. kefiri* KFLM3 adsorbed 80–100% of AFB1, ZEA, and OTA when cultivated in milk [170]. The adsorption mechanism involves cell wall components, particularly peptidoglycan and proteins, with hydrophobicity and surface roughness influencing binding capacity [136].

In food matrix applications (contaminated oat and wheat substrates), fermentation with LAB starter cultures reduced T-2 toxin concentrations by 20–45% compared to non-fermented controls, with reductions partially attributable to the antifungal activity of LAB against *F. sporotrichioides* [51]. Cell wall adsorption efficiencies for T-2 toxin by selected LAB strains range from 20–50%, with *L. rhamnosus* and *L. plantarum* demonstrating the most consistent adsorption.

## 6. Application of LAB Detoxification in Food Systems

### 6.1. Cereal and Bread Fermentation

**Figure 2:**
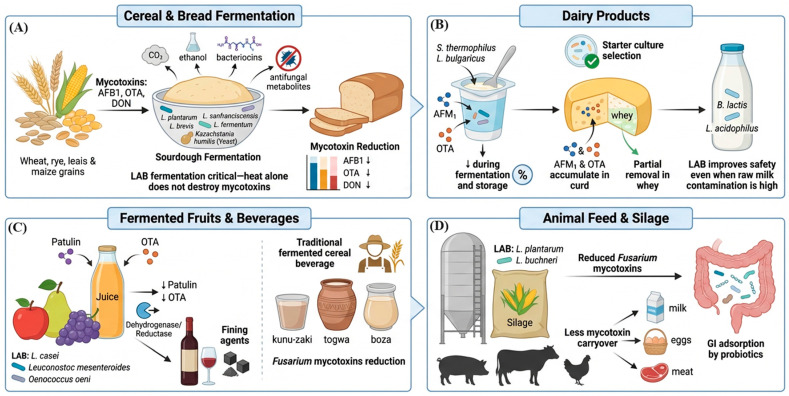
Detoxification of mycotoxins by LAB in food and feed systems. (**A**) Detoxification in cereals and bread fermentation, (**B**) detoxification in dairy products, (**C**) detoxification in fermented fruits and beverages, and (**D**) detoxification in animal feed and silage.

Sourdough fermentation is one of the most extensively studied food systems for LAB-mediated mycotoxin control (Figure 2A). Sourdough is a leavened dough fermented by a complex microbiome of LAB (principally *L. plantarum*, *Fructilactobacillus sanfranciscensis*, *L. brevis*, and *L. fermentum*) and sourdough-adapted yeasts (*Kazachstania humilis*, formerly *Candida humilis*). *Fructilactobacillus sanfranciscensis* (formerly *Lactobacillus sanfranciscensis*) is consistently identified as the key autochthonous LAB species in traditional sourdoughs and is described as “sourdough adapted” [171,172]. The rapid acidification to pH 3.8–4.5, combined with CO_2_, ethanol, bacteriocins, and a range of antifungal metabolites, creates multiple barriers to toxigenic fungal growth and mycotoxin persistence [86,122]. Research demonstrates that sourdough fermentation and lactic acid bacteria effectively reduce mycotoxin levels in cereal-based products. Escrivá et al. [24] found that *Lactobacillus* strains reduced AFB1 by up to 55% and OTA by up to 34% in bread compared to controls. Similarly, Lafuente et al. [173] showed that dried sourdough significantly reduced aflatoxin contamination, with *P. pentosaceus* TI6-fermented sourdough achieving the lowest levels. Pakfetrat et al. [174] reported germination reduced DON by 39%, OTA by 38%, and various aflatoxins by 33–62% after 10–14 days. Meta-analyses by Mousavi Khaneghah et al. [175] and Schaarschmidt & Fauhl-Hassek [176] confirmed that fermentation generally reduces OTA and ZEN concentrations, while affecting DON and aflatoxins variably depending on processing conditions. Wang et al. [176] and Deligeorgakis et al. [177] documented widespread mycotoxin contamination in wheat flours, emphasizing the importance of processing interventions. Escrivá et al. [178] further demonstrated that fermented ingredients reduce mycotoxin bioaccessibility by 57–74% for AFB1 and 11–34% for OTA.

### 6.2. Dairy Products

Lactic acid bacteria demonstrate significant potential to reduce mycotoxin levels in dairy products through multiple mechanisms (Figure 2B). Several studies confirm that LAB strains can effectively bind AFM1 in yogurt and cheese systems, with binding capacities ranging from 49% to 61% [179]. Heat-killed LAB cells maintain mycotoxin adsorption abilities, achieving up to 100% AFM1 reduction in Frescal cheese during storage [33]. Beyond AFM1, LAB strains exhibit broad-spectrum mycotoxin-binding capabilities, reducing aflatoxin B1, ochratoxin A, and zearalenone by 11–55% across various food matrices [24,104,180]. Kefir-derived microorganisms, particularly *Lactobacillus kefiri*, demonstrate exceptional mycotoxin adsorption rates of 80–100% when cultivated in milk [170]. The mechanisms involve both adsorption and biodegradation processes, with viable cells generally showing superior performance compared to heat-inactivated cells [104]. These findings support the use of LAB as bioprotective cultures for preserving dairy products [181].

### 6.3. Fermented Fruits and Beverages

Apple cider fermentation and direct LAB treatment of apple juice are practical strategies for reducing patulin in fruit products through multiple mechanisms (Figure 2C). Zheng et al. [100] showed that *Lactobacillus casei* YZU01 completely degraded 10 μg/mL patulin in raw apple and pear juices within 36–48 h through extracellular metabolite secretion and cell wall adsorption. Similarly, Ngolong Ngea et al. [182] found that *Leuconostoc mesenteroides* subsp. *mesenteroides* LB7 successfully detoxified both homemade and commercial fruit juices contaminated with patulin. Bahati et al. [101] demonstrated that inactivated *Lactobacillus kefiranofacien* achieved 93% patulin removal from apple juice through adsorption mechanisms involving C=O, OH, C–H, and N–O functional groups. Li et al. [183] emphasized that LAB-mediated degradation and patulin adsorption require further investigation as part of comprehensive patulin mitigation strategies. Enzymatic approaches also show promise, with Xing et al. [164] reporting that a short-chain dehydrogenase/reductase reduced patulin by 80% in apple juice while maintaining product quality. Research demonstrates that *Lactobacillus* strains effectively reduce OTA contamination during grape juice fermentation and wine production through multiple mechanisms. *Lactobacillus rhamnosus* Bm01 removed 83.87% of OTA from grape juice within 48 h, primarily through cell wall adsorption [138]. Similarly, thiol-modified *Lactobacillus plantarum* achieved >90% OTA removal in grape juice [184]. *Lactobacillus* strains can be successfully incorporated as starter cultures in wine production, offering the dual benefits of promoting malolactic fermentation and reducing OTA contamination [137]. Fermented cereal beverages (African kunu-zaki, togwa, boza) naturally contain abundant LAB communities that reduce *Fusarium* mycotoxins (DON, FB1, ZEA) during their preparation. Ethnographic and microbiological studies from Nigeria, Tanzania, and Turkey have documented that traditional fermentation practices involving LAB can reduce fumonisin and DON by 50–70% compared to unfermented grain, providing evidence that traditional food knowledge aligns with modern mycotoxin management principles [84,185].

### 6.4. Animal Feed and Silage

While this review focuses on human food systems, LAB applications in animal feed silage are briefly covered, given the feed-to-food relevance via animal products (Figure 2D). Silage fermentation with LAB inoculants (*L. plantarum*, *L. buchneri*) dramatically reduces the risk of *Fusarium* mycotoxin accumulation in high-moisture maize and grass silage by competing with mycotoxigenic fungi during ensiling [186]. Probiotic LAB supplementation effectively reduces mycotoxin bioavailability in poultry and swine feed through multiple mechanisms. LAB strains demonstrate significant mycotoxin removal capabilities, with *L. acidophilus* and *L. delbrueckii* reducing AFB1 by 33% and ZEA by 28% in laboratory conditions [104]. In broiler studies, probiotic supplementation containing multiple LAB strains significantly increased AFB1 excretion and reduced AFB1 residues in liver and kidneys by approximately 58–70% [131].

## 7. Toxicity Profiles of Major Mycotoxin Degradation Products

The safety of degradation products generated during LAB-mediated mycotoxin biotransformation is a critical yet frequently underreported dimension of biodetoxification research, and the rigor of the available evidence varies substantially across mycotoxin classes (Table 3). For AFB1, *L. helveticus* FAM22155 produced four lactone-ring-open metabolites identified by LC-MS [187]; because the lactone ring is central to AFB1 genotoxicity, its absence implies lower toxicological potential, but no formal cytotoxicity assays were conducted, and reduced toxicity therefore remains structurally inferred. An alternative LAB-associated mechanism, thermal treatment of AFB1 with lactic acid (a metabolite of LAB) at 80 °C, yields AFB2 and AFB2a, with AFB2a showing markedly reduced cytotoxicity in the HeLa MTT assay [188], although this reaction depends on heat rather than enzymatic activity and is unlikely to occur under standard fermentation conditions. For OTA, amide bond hydrolysis by LAB carboxypeptidase or amidohydrolase yields an OTα and l-β-phenylalanine, confirmed by LC-MS [189]; OTα is substantially less nephrotoxic than OTA and exhibits reduced albumin-binding affinity, yet retains residual mutagenic and phytotoxic activity [98], and long-term in vivo safety data remain absent. For PAT, the evidence base is the most complete: *L. plantarum* 13M5 biotransforms PAT to (E)-ascladiol via enzymatic ring opening, an activity confined to living cells, thereby reducing PAT-induced cytotoxicity and intestinal barrier disruption in Caco-2 cells [190]. Both E- and Z-ascladiol are devoid of cytotoxicity across human liver, kidney, intestinal, and immune cell lines, and microarray analysis showed no transcriptomic perturbation by E-ascladiol [165]. Supporting in vivo evidence from mice showed no organ toxicity, intestinal damage, or microbiota disruption following exposure to *L. casei* YZU01-derived PAT degradation products [191], making PAT-to-ascladiol biotransformation one of the most robustly validated detoxification pathways in the LAB literature. The toxicological outcome of LAB biotransformation of ZEA is pathway-dependent and unpredictable without metabolite-specific profiling. Carbonyl reductase-mediated conversion by *L. paracasei* 85 and *L. buchneri* 93 yielded β-ZOL at >2-fold the level of α-ZOL [105], which is desirable since β-ZOL is less estrogenic than ZEA; conversely, strains that predominantly produce α-ZOL, as observed for fermentative LAB in simulated silage [192], may perpetuate or exacerbate estrogenic risk. The benchmark detoxification endpoint, HZEN and DHZEN produced by lactonase Zhd101p, is 50–10,000-fold less estrogenic than ZEA in vitro and elicited no uterotrophic effects in piglets [193], providing a clear toxicological target for future strategies. For DON, the benchmark degradation products DOM-1 and 3-epi-DON form only two hydrogen bonds with the ribosomal A-site (vs. three for DON), abolishing MAPKinase activation; neither metabolite impaired intestinal barrier function in human epithelial models or caused lesions in piglets following seven-day exposure at 3 mg/kg [194,195], although for LAB, adsorption remains the dominant DON reduction mechanism. For FB1, *Lactobacillus* sp. from silage produced hydrolyzed FB1 (HFB1) as an intermediate during FB1 degradation [196]; benchmark toxicology confirms that HFB1 does not cause hepatotoxicity, does not inhibit ceramide synthase, and only weakly perturbs the sphinganine/sphingosine ratio in a two-week piglet study [197], supporting HFB1 as a safer metabolite, although food-matrix validation is still required. Taken together, these findings underscore that degradation product safety cannot be inferred from removal efficiency alone and must be assessed on a case-by-case basis for each mycotoxin-LAB pairing; regulatory acceptance of any LAB-based detoxification strategy will require formal toxicological dossiers for all biotransformation products before food-scale application can be justified [198,199].

## 8. Factors Governing LAB Mycotoxin Detoxification Efficiency

### 8.1. Strain-Specific and Species-Level Variation

Inter-strain variation in mycotoxin binding and degradation capacity within a single species can exceed inter-species differences, making strain-level screening essential for identifying superior detoxification agents [83]. Studies comparing multiple strains of *L. rhamnosus* for AFB1 binding reveal binding efficiencies of 10–90% among strains of the same species, attributable to differences in cell wall composition, EPS production, surface hydrophobicity, and enzyme complement [74,77]. The genetic basis for this variation is being elucidated through comparative genomics of detoxification-competent versus non-competent LAB strains, with cell wall biosynthesis genes, teichoic acid modification loci, and metabolic enzyme clusters showing the strongest associations with detoxification phenotype [200].

### 8.2. Mycotoxin Concentration and Structural Class

The efficiency of LAB mycotoxin binding generally decreases as initial toxin concentration increases, suggesting saturation of available binding sites on bacterial surfaces following Langmuir isotherm kinetics [80]. Highly lipophilic mycotoxins (AFB1, OTA, ZEA) with aromatic ring systems exhibit stronger adsorption onto bacterial surfaces than polar trichothecenes (DON) lacking significant hydrophobic domains [40]. Molecular weight, molecular planarity, and the presence of polar functional groups capable of hydrogen bonding collectively influence the specificity and strength of LAB-mycotoxin interactions [80,89].

### 8.3. Environmental and Process Conditions

Lactic acid bacteria demonstrate significant potential for mycotoxin detoxification through binding and biotransformation mechanisms. Multiple studies show that LAB can effectively remove various mycotoxins, including AFB1, OTA, ZEA, and DON, with strain-specific efficiencies ranging from 16–71% for AFB1 [201]. pH emerges as a critical modulating factor, with removal efficiency of viable LAB cells increasing as pH decreases from 6 to 5 [202]. Temperature effects are generally modest within the 4–37 °C range relevant to food fermentation and storage, but extreme temperatures affecting bacterial cell wall integrity can alter binding kinetics [203]. Ionic strength and the composition of the food matrix (proteins, lipids, carbohydrates) influence binding competition, as components of the food matrix may compete for binding sites or protect mycotoxins through matrix interactions [204].

### 8.4. Viable vs. Non-Viable (Heat-Killed) Cells

Research demonstrates that LAB effectively binds mycotoxins, with non-viable cells often showing superior performance compared to viable cells. Heat-killed and acid-killed LAB cells consistently exhibit enhanced mycotoxin-binding capacity for aflatoxin B1 (AFB1) and ochratoxin A (OTA) [201,202,205]. This improved binding is attributed to cell membrane denaturation that exposes additional internal binding sites upon inactivation [206]. Studies show AFB1 removal rates of 16–71% depending on strain and cell viability, with non-viable cells demonstrating superior performance [201]. Thermosonication treatment further enhances binding capacity compared to heat treatment alone [206]. However, some studies report contradictory findings, with heat-inactivated cells producing significantly lower reductions in mycotoxin levels than viable cells under certain conditions [104]. The binding mechanism appears to be the primary mode of mycotoxin reduction rather than biodegradation [201,207].

### 8.5. Fermentation Duration and Inoculum Density

Extended fermentation times generally improve mycotoxin reduction efficiencies up to an optimum, after which toxin re-release or metabolic transformation may cause apparent reduction in efficacy [28]. Higher LAB inoculum densities provide more binding sites per unit volume and more rapid acidification, both of which contribute to enhanced mycotoxin reduction. Industrial applications targeting 10^8^–10^10^ CFU/mL inoculum densities in sourdough fermentations achieve substantially greater reductions in AFB1 and OTA than lower-density inocula [86,122].

## 9. In Vivo Evidence and Safety of Degradation Products

Translating in vitro LAB detoxification data to in vivo settings is essential but challenging due to the complex interactions among LAB, the food matrix, the gut microbiota, intestinal transit, and mycotoxin metabolism. Multiple animal model studies have now confirmed the protective efficacy of LAB against mycotoxin-induced toxicity, providing the mechanistic rationale for clinical applications. In broiler chickens, *Lactobacillus*-based probiotics significantly reduced AFB1 residues in liver and kidney tissues by 58–70% [131,208]. These interventions improved growth performance and feed conversion ratios while reducing serum liver enzymes (AST, ALT), indicative of hepatoprotection [208]. Rat model studies by Gratz et al. [126] using gavage-administered *L. rhamnosus* GG demonstrated significantly reduced AFB1-DNA adduct formation in liver tissue and increased fecal AFB1 excretion, confirming gastrointestinal adsorption as the in vivo mechanism.

Ochratoxin A (OTA) is a nephrotoxic mycotoxin produced by *Aspergillus* and *Penicillium* fungi that contaminates food and feed, causing kidney damage, oxidative stress, and inflammation in animals [209,210]. Pigs are particularly susceptible to OTA nephrotoxicity, with studies demonstrating accumulation in kidney tissue and histopathological lesions following dietary exposure [211,212]. Biological detoxification methods using microorganisms show promise for OTA removal, with the primary mechanism being hydrolysis of the amide bond to produce ochratoxin α (OTα) [98]. While OTα is substantially less toxic than OTA, it retains some genotoxic potential [213]. OTA exposure causes multiple adverse health effects, including genotoxicity, nephrotoxicity, and potential carcinogenicity, with mechanisms involving DNA damage, oxidative stress, and inhibition of protein synthesis [214,215,216].

In vivo reduction of DON-induced intestinal permeability disruption by LAB supplementation has been demonstrated in piglets, with *L. plantarum* reducing DON-induced production of pro-inflammatory cytokines (IL-6, TNF-α) and improving tight junction protein expression in the intestinal epithelium [217].

Multiple studies demonstrate that patulin degradation products, particularly E-ascladiol and desoxypatulinic acid (DPA), exhibit significantly reduced cytotoxicity compared to patulin itself. Tannous et al. [165] showed that both E- and Z-ascladiol were completely non-cytotoxic against human liver, kidney, intestinal, and immune system cell lines, whereas patulin exhibited dose-dependent cytotoxicity. Similarly, Zheng et al. [218] found that ascladiol degradation products were significantly less toxic to *E. coli*, *Arabidopsis thaliana*, and human esophageal epithelial cells compared to patulin. Ianiri et al. [219] confirmed that both desoxypatulinic acid and ascladiol were less toxic than patulin, while Pinedo et al. [220] specifically demonstrated that DPA caused much lower chromosomal damage than patulin in human lymphocytes.

Research demonstrates that LAB can effectively remove zearalenone (ZEA) via multiple mechanisms, but the estrogenic potency of the resulting metabolites varies widely. Several Lactobacillus strains, including *L. plantarum*, *L. paracasei*, and *L. buchneri*, achieve ZEA removal rates of 50–78% through adsorption and biotransformation [102,103,105]. The primary removal mechanisms involve hydrophobic interactions with cell wall components and enzymatic degradation [103,104]. Critically, ZEA metabolites exhibit markedly different estrogenic activities. Hydrolyzed zearalenone (HZEN) and decarboxylated hydrolyzed zearalenone (DHZEN) show 50–10,000 times reduced estrogenicity compared to ZEA [193]. Similarly, phosphorylated ZEA conjugates demonstrate reduced estrogenic toxicity [221]. Furthermore, the ZEA reduction products, α- and β-zearalenol, were detected in LAB cultures [105], highlighting the importance of metabolite-specific analysis when evaluating LAB strains for ZEA detoxification applications [222,223]. Regulatory guidance on acceptable degradation product profiles is needed and is under development by EFSA and international bodies.

## 10. Regulatory Considerations for LAB-Based Detoxification

The regulatory approval pathway for LAB-based mycotoxin detoxification agents depends on the intended application: as a food ingredient, processing aid, probiotic supplement, or biocontrol agent for pre-harvest use. In the European Union, biological detoxification agents for aflatoxin and OTA control in feed are regulated under Regulation (EC) No 1831/2003 on feed additives, and applications require demonstration of safety, efficacy, and non-adverse environmental impact [224]. The approval process requires characterization of the active substance, the targeted mycotoxin, the applicable food/feed category, the proposed use level, and a full toxicological dossier, including metabolite safety data.

For human food applications, LAB-based detoxification agents must comply with food safety legislation (Regulation (EC) No 178/2002; Regulation (EC) No 1333/2008 for food additives; Novel Food Regulation (EU) 2015/2283 for novel food ingredients or new processing technologies). LAB strains with established QPS or GRAS status benefit from a simplified regulatory pathway but must demonstrate their safety and efficacy for the specific mycotoxin and food matrix in question [67]. The use of metabolically active LAB (containing CRISPR-engineered or recombinant enzyme activities) triggers additional requirements under EU GMO legislation. Codex Alimentarius, through the Joint FAO/WHO Expert Committee on Food Additives (JECFA) and the Codex Committee on Contaminants in Foods (CCCF), provides international guidance on maximum mycotoxin levels and acceptable control measures. The incorporation of LAB detoxification into Good Manufacturing Practice (GMP) frameworks as a validated control measure (equivalent to the Physical Control Measure in HACCP) would represent a significant regulatory advancement, but it would require standardized protocols, validated analytical methods, and collaborative harmonization among national food safety authorities [29,31,225].

## 11. Challenges, Emerging Strategies, and Future Perspectives

### 11.1. Current Limitations

Despite decades of research, several challenges impede the widespread adoption of LAB-based mycotoxin detoxification in the food industry. First, the high inter-strain variability in detoxification capacity necessitates extensive screening programs for each new food application, as no universal high-performance strain has been identified that effectively detoxifies all major mycotoxin classes simultaneously [83]. Second, most published studies use model systems (MRS broth, phosphate-buffered saline) that may not accurately predict performance in complex food matrices where matrix-mycotoxin and matrix-LAB interactions alter both toxin bioavailability and bacterial behavior [204]. Third, the reversibility of LAB-mycotoxin adsorption complexes in the gastrointestinal tract remains incompletely characterized; if bound mycotoxins are released in sufficient quantities under GI conditions, apparent in vitro detoxification may not translate to reduced human exposure [90]. Fourth, regulatory frameworks for LAB-based detoxification agents lack international harmonization, creating barriers to market access for food producers seeking to use validated strains across jurisdictions [29,31,225]. Fifth, the safety assessment of degradation products—particularly for enzymatic biotransformation is incomplete for most mycotoxin-LAB pairings. Various microbial enzymes, including oxidoreductases and hydrolases, have been identified for mycotoxin biotransformation, with expression systems ranging from traditional *E. coli* and yeasts to novel platforms like *Bacillus subtilis* and LAB [198,226]. However, significant gaps in safety assessment remain, particularly regarding degradation products of enzymatic pathways [198]. While LAB show promise for *Fusarium* mycotoxin detoxification [78], systematic safety evaluation of both biosynthetic enzymes and their degradation products is urgently needed for commercial applications [198]. Current limitations include incomplete toxicological data for many mycotoxin-enzyme pairings and insufficient understanding of degradation mechanisms [56,199].

### 11.2. Encapsulation and Strain Engineering

Microencapsulation of LAB in protective matrices (alginate, chitosan, whey protein, starch) enhances bacterial viability during food processing and gastrointestinal transit, potentially improving the efficiency of in vivo mycotoxin sequestration [227]. Encapsulated LAB preparations show enhanced survival under conditions (high temperature, acidic pH, oxygen exposure) that are inimical to free-cell viability, expanding the range of food products in which LAB detoxification agents could be incorporated [228]. Genetic engineering of LAB to express mycotoxin-degrading enzymes (OTA-hydrolyzing carboxypeptidases, ZEA-specific lactonases, AFB1-transforming oxidoreductases) holds transformative potential to enhance and broaden detoxification capabilities [200,229]. Using GRAS LAB as chassis organisms, heterologous expression of characterized mycotoxin-degrading enzymes from other organisms (fungi, bacteria, soil microbiomes) into food-compatible LAB strains could create highly efficient, multi-mycotoxin-degrading starter cultures. Regulatory acceptance of such strains will require a comprehensive safety assessment and transparent labeling requirements.

### 11.3. Multi-Target and Combination Approaches

Single-mechanism approaches (adsorption alone or enzymatic biotransformation alone) are insufficient against the complex, multi-mycotoxin contamination profiles encountered in real food commodities. Integrated approaches combining LAB with other biological agents (yeasts, *Bacillus* spp.), physical treatments (UV radiation, cold plasma), chemical agents (organic acids, plant extracts), and biocontrol agents (antagonistic fungi, mycoparasites) demonstrate synergistic effects that exceed the sum of individual strategies [53,230]. The field of systems mycology and multi-omics approaches (transcriptomics, proteomics, metabolomics) applied to LAB-mycotoxin interactions is beginning to reveal regulatory networks governing detoxification capacity, suggesting targets for rational strain improvement [78]. Machine learning and artificial intelligence approaches applied to large screening datasets may accelerate the identification of optimal strain-mycotoxin-food matrix combinations, reducing the time and cost of empirical screening programs [152,231].

Precision fermentation is an advanced biotechnological approach that uses genetically modified microorganisms to produce specific food ingredients with greater efficiency and sustainability [232,233]. This field integrates cutting-edge techniques, including CRISPR-Cas9 genome editing, metabolic engineering, and AI-guided strain optimization to improve fermentation processes [234,235]. Multi-omics approaches, particularly metagenomics, transcriptomics, and metabolomics, provide comprehensive insights into microbial diversity and metabolic pathways, enabling targeted biocontrol strategies [236,237]. These technologies show particular promise for mycotoxin detoxification via microbial mechanisms, including enzymatic biodegradation and cell wall adsorption [236]. Emerging technologies like ultrasound, pulsed electric fields, and cold plasma can be combined with fermentation to enhance mycotoxin degradation [238]. Traditional fermentation already serves as an effective mycotoxin decontamination strategy, particularly valuable in resource-limited settings [239].

### 11.4. Climate Change and Emerging Mycotoxins

Climate change represents an emerging driver of mycotoxin risk in food systems, with projections of northward expansion of aflatoxin-producing fungi in Europe, increased fumonisin occurrence in temperate cereal zones, and novel mycotoxin profiles associated with warming-induced shifts in fungal community composition [15,240]. LAB-based detoxification strategies need to be evaluated for efficacy against emerging mycotoxins (enniatins, beauvericin, alternariol, altertoxins) that are increasingly detected in European monitoring programs [55]. The rapid development of climate-adaptive LAB starter cultures capable of broader-spectrum mycotoxin management represents a proactive approach to future food safety challenges.

## 12. Conclusions

Biological detoxification of mycotoxins using LAB is a scientifically validated, practically applicable, and regulatory-compatible strategy to enhance food safety amid global mycotoxin contamination. The multiple mechanisms by which LAB reduce mycotoxin bioavailability, physical adsorption onto cell wall components, enzymatic biotransformation to less toxic products, antifungal metabolite-mediated inhibition of toxigenic fungi, and competitive ecological exclusion, provide a multi-barrier approach that is particularly well suited for integration into existing food fermentation processes. The evidence base is most robust for aflatoxin B1 and M1, ochratoxin A, and patulin, where both in vitro and food-matrix studies corroborate the in vivo protective effects observed in animal models. Detoxification of trichothecenes (DON, T-2), fumonisins, and zearalenone, while demonstrated in model systems, requires further optimization and in vivo validation. Strain-specific performance variability necessitates systematic screening programs for industrial strain selection, with emerging genomic tools enabling more rational and rapid identification of high-performance detoxification candidates. Key challenges to widespread adoption include: standardization of detoxification assays and performance benchmarks; safety characterization of biotransformation products; regulatory harmonization across jurisdictions; and validation of efficacy in complex food matrices at an industrial scale. Future research should prioritize mechanistic safety characterization of biotransformation products, the development of metabolically engineered food-grade LAB with broadened detoxification spectra, encapsulation strategies to enhance process stability, and clinical evidence supporting protection of human populations against dietary mycotoxin exposure. Integrating LAB-based detoxification with other food safety control measures within a comprehensive HACCP framework holds the greatest promise for meaningful, scalable reduction of mycotoxin contamination in the global food supply.

## Figures and Tables

**Table 1:** Major food mycotoxins of regulatory significance: producing fungi, affected commodities, health effects, and EU/Codex maximum limits.

Mycotoxin	Producer Fungi	Affected Commodities	Primary Health Effects	EU Limit (µg/kg)	Codex/FDA (µg/kg)	References
Aflatoxin B1 (AFB1)	*Aspergillus flavus*, *A. parasiticus*	Maize, peanuts, tree nuts, spices, dried figs, cottonseed	Hepatocarcinogen (Group 1, IARC); immunosuppressive; mutagenic; teratogenic; growth retardation	2 (cereals); 10 (nuts)	20 total AFs (FDA); 0.5 AFM1 (Codex)	[30,31,32]
Aflatoxin M1 (AFM1)	Metabolite of AFB1 in lactating animals fed contaminated feed	Milk, yogurt, cheese, butter, infant formula	Hepatocarcinogen (Group 2B, IARC); immunosuppressive; detected in breast milk	0.05 (milk)	0.5 milk (Codex)	[32,33,34]
Ochratoxin A (OTA)	*Aspergillus ochraceus*, *A. carbonarius*, *Penicillium verrucosum*	Cereals, wine, grape juice, dried fruits, coffee, cocoa, spices	Nephrotoxic; immunosuppressive; probable carcinogen (Group 2B, IARC); teratogenic; kidney tumors in rodents	3 (cereals); 10 (dried grapes)	No Codex limit; country-specific	[32,35,36,37]
Deoxynivalenol (DON)	*Fusarium graminearum*, *F. culmorum*	Wheat, barley, maize, oats; bread, beer, pasta, breakfast cereals	Ribotoxic stress response; immunotoxic; gastrointestinal toxicity; anorexia; growth retardation; intestinal barrier disruption	750–1250 (cereals); 200 (infant food)	1000 µg/kg (WHO); 1 mg/kg advisory (FDA)	[38,39,40,41,42]
Zearalenone (ZEA)	*Fusarium graminearum*, *F. culmorum*, *F. equiseti*	Maize, wheat, barley, sorghum, processed cereal products	Mycoestrogen; binds ERα and ERβ; reproductive toxicity; hyperestrogenism in swine; endocrine disruption	100–350 (cereals); 20 (infant food)	No Codex limit; country-specific	[32,43,44]
Fumonisins B1/B2 (FB1/FB2)	*Fusarium verticillioides*, *F. proliferatum*	Maize, sorghum, wheat; processed maize products, tortillas	Inhibits ceramide synthase; equine leukoencephalomalacia; porcine pulmonary edema; esophageal cancer risk (Group 2B, IARC)	200–4000 (cereals); 800–1000 (maize flour)	2 mg/kg maize (FDA); no Codex limit	[45,46]
Patulin (PAT)	*Penicillium expansum*, *Aspergillus clavatus*, *Byssochlamys* spp.	Apple juice, apple products, pear juice, fruit-based baby foods	Reacts with thiol groups; gastrointestinal and neurological toxicity; genotoxic; immunosuppressive	10–50 (fruit juices/products)	50 µg/kg apple juice (Codex)	[47,48,49]
T-2/HT-2 Toxin	*Fusarium sporotrichioides*, *F. langsethiae*, *F. poae*	Oats, wheat, maize, barley, rye; cereal products	Protein and DNA synthesis inhibition; radiomimetic effects; immunosuppression; alimentary toxic aleukia; acute dermotoxicity	100–200 indicative (EFSA guidance)	No Codex limit established	[40,50,51,52]
Citrinin (CIT)	*Penicillium citrinum*, *Monascus purpureus*, *Aspergillus niveus*	Cereals, red yeast rice, dried beans, food supplements	Nephrotoxic; hepatotoxic; genotoxic; often co-occurs with OTA (synergistic nephrotoxicity)	100 (food supps., EFSA guidance)	No Codex limit	[16,37,53,54]
Enniatins/Beauvericin	*Fusarium tricinctum*, *F. avenaceum*, *Beauveria bassiana*	Wheat, maize, barley, cereal-based products	Emerging mycotoxins; ionophore activity; cytotoxic; disrupts membrane ion homeostasis; increasing occurrence in EU cereals	Under evaluation	No established limits	[54,55,56]

**Table 2:** Mycotoxin detoxification by several species of LAB in different food samples.

LAB Species	GRAS/QPS	Food Applications	Mycotoxins Targeted (Removal %)	Primary Mechanism(s)	References
*Lactiplantibacillus plantarum*	QPS (EFSA); GRAS (FDA)	Sourdough, fermented vegetables, silage, fermented meats, probiotics	AFB1 (40–90%); OTA (30–60%); DON (20–45%); ZEA (65–90%); PAT (50–90%); FB1 (10–40%); T-2 (20–40%)	Cell wall adsorption (peptidoglycan, teichoic acids); antifungal metabolites (phenyllactic acid (PLA), lactic acid); competitive exclusion	[21,24,26,41,74,75,76]
*Lacticaseibacillus rhamnosus*	QPS (EFSA); GRAS (FDA)	Probiotic supplements, fermented dairy, yogurt, cheese	AFB1 (60–90%); AFM1 (30–71%); OTA (15–60%); DON (20–55%); T-2 (20–50%)	Surface adsorption (EPS; peptidoglycan); GI-tract sequestration; adsorption/desorption kinetics well characterized	[33,40,74,77,78]
*Lacticaseibacillus casei*	QPS; GRAS	Cheese (e.g., Cheddar), fermented milk, probiotic beverages	AFB1 (30–70%); AFM1 (40–71%); OTA (20–55%); DON (15–40%); PAT (30–65%)	Cell wall adsorption; carboxypeptidase-mediated OTA hydrolysis (OTα); thiol adduction of PAT	[24,79,80]
*Limosilactobacillus reuteri*	QPS; GRAS	Probiotic supplements, fermented dairy, sourdough	AFB1 (25–60%); OTA (20–50%); DON (15–40%); ZEA (20–50%)	Cell wall adsorption; reuterin production (antifungal); competitive exclusion in fermentation matrices	[33,36,40,81]
*Lactobacillus acidophilus*	QPS; GRAS	Yogurt, probiotic dairy, dietary supplements, fermented cereal	AFB1 (30–75%); AFM1 (25–65%); OTA (15–55%); ZEA (25–60%); DON (15–40%)	Surface adsorption; EPS-enhanced binding; carboxypeptidase activity (OTA hydrolysis); antifungal organic acid production	[21,24,33,80,82]
*Lactobacillus fermentum*	QPS; GRAS	Fermented cereals (ogi, kunu), sourdough, African fermented foods	AFB1 (25–65%); DON (15–50%); FB1 (10–35%); OTA (15–40%)	Cell wall adsorption; organic acid and hydrogen peroxide production; antifungal inhibition of Fusarium and Aspergillus	[40,41,83,84]
*Lactococcus lactis*	QPS; GRAS	Dairy fermentation (cheese, butter), nisin production, soft cheeses	OTA (20–55%); ZEA (20–50%); DON (15–40%); PAT (30–60%)	Cell wall adsorption; nisin-mediated antifungal activity; protease secretion (OTA amide bond cleavage)	[21,36,69]
*Leuconostoc mesenteroides*	QPS; GRAS	Sauerkraut, kimchi, fermented vegetables, sourdough, fermented beverages	ZEA (15–45%); DON (10–35%); OTA (10–30%)	Cell wall adsorption; competitive exclusion; acidification via heterofermentation (lactic + acetic acid)	[40,81,85,86]
*Pediococcus acidilactici*	QPS; GRAS	Fermented meats (salami), dry sausages, vegetable fermentation, probiotic feeds	AFB1 (20–50%); OTA (15–45%); DON (10–35%); ZEA (15–45%)	Cell wall adsorption; pediocin production (antifungal); EPS-mediated binding in fermented substrates	[21,33,40,81,83]
*Streptococcus thermophilus*	QPS; GRAS	Yogurt starter (with *L. delbrueckii* subsp. *bulgaricus*), mozzarella, fermented milks, thermophilic cheese	AFB1 (20–55%); AFM1 (22–50%); OTA (15–40%)	Surface adsorption; EPS production; dairy-matrix-specific binding during coagulation; acidification	[33,82,87]
*Enterococcus faecium*	Not on EFSA QPS list (excluded from QPS evaluation per 2025 EFSA update; individual strain safety assessment required before use)	Selected probiotic strains; ripened cheese, fermented meat; silage	AFB1 (15–45%); OTA (15–45%); DON (10–30%)	Cell wall adsorption (peptidoglycan, polysaccharide); AFB1 binding via cell wall amide groups; limited enzymatic degradation	[40,68,81,88,89]
*Bifidobacterium* spp.	QPS; GRAS	Probiotic dairy (yogurt, kefir), fermented milk, infant formula supplements	AFB1 (25–65%); AFM1 (20–60%); OTA (15–50%); DON (10–35%)	EPS-enhanced surface adsorption; *Bifidobacterium*-specific cell wall polysaccharide binding; GI-tract sequestration in vivo	[33,67,80,82,90]
*Oenococcus oeni*	QPS (EFSA, wine-specific)	Malolactic fermentation in wine, cider, and fruit wine	OTA (15–40%); PAT (20–50%)	Cell wall adsorption during malolactic fermentation; malic acid-driven substrate changes reduce toxin stability	[28,33,91]
*Weissella* spp.	QPS; GRAS (selected spp.)	Fermented cereals; kimchi; fermented fish; sourdough (*W. confusa*)	AFB1 (15–40%); ZEA (10–35%); OTA (10–30%)	EPS-mediated adsorption; antifungal metabolite secretion; sourdough acidification reducing toxigenic mold growth	[21,23,83,86,92]

**Table 3:** Toxicity profiles of major mycotoxin degradation products generated during LAB-mediated detoxification.

Parent Mycotoxin	LAB/Mechanism	Main Degradation Product(s)	Toxicity vs. Parent Toxin	Remarks	References
AFB1	*L. helveticus* FAM22155; extracellular protein fraction; solid-state fermentation; biotransformation	Four lactone-ring-open products (by LC-MS)	Lower toxicity inferred structurally (lactone ring absent)	No formal cytotoxicity data; toxicity reduction inferred from structure only	[187]
	Lactic acid (LAB metabolite); heating at 80 °C	AFB2 and AFB2a	AFB2a much less cytotoxic than AFB1 (HeLa MTT assay)	Chemical degradation by lactic acid (not LAB enzymatic); requires heating	[188]
OTA	LAB: enzymatic degradation + cell wall adsorption	OTα + l-β-phenylalanine	OTα confirmed by MS; direct OTα vs. OTA comparison not within the same study	OTα has reduced nephrotoxicity and albumin-binding vs. OTA (literature); residual mutagenic potential reported; long-term safety data absent	[189]
PAT	*Lactiplantibacillus plantarum* 13M5; biotransformation (living cells only)	(E)-ascladiol	Reduced cytotoxicity and barrier disruption vs. PAT in Caco-2 cells	Living cells required; heat-killed cells did not degrade PAT; degradation up to 43.81%	[190]
	Lactobacilli screened in apple juice; biotransformation + thiol-adduct formation	(E)-ascladiol; Z-ascladiol (traces)	E- and Z-ascladiol are devoid of cytotoxicity across human liver, kidney, intestinal, and immune cell lines	Ascladiol did not alter the human transcriptome (microarray); patulin detoxification strategies producing ascladiol are considered safe	[165]
ZEN	*L. paracasei* 85 + *L. buchneri* 93; binding + biotransformation	α-ZOL and β-ZOL	Mixed: β-ZOL formed >2× α-ZOL; β-ZOL is less estrogenic than α-ZOL, net estrogenicity reduced	Metabolite ratio critical: α-ZOL may be more estrogenic than ZEN itself; product profiling essential for any ZEN-detoxifying strain	[105]
	Fermentative bacteria (8 Lactobacilli, 3 *Leuconostoc*) in simulated silage	α-zearalenol (α-ZOL)	α-ZOL can be more estrogenic than ZEN	No DON or fumonisin biotransformation detected; only adsorption for those; ZEN biotransformation outcome strain-dependent	[192]
	ZEN lactonase Zhd101p (benchmark; non-LAB enzyme)	HZEN and DHZEN	50–10,000× less estrogenic than ZEN in vitro; no uterotrophic effect in piglets	Benchmark evidence: enzyme is non-LAB. HZEN/DHZEN represent the preferred detoxification endpoint for ZEN; lactonase-producing LAB under investigation	[193]
DON	Non-LAB bacterial transformation (benchmark)	DOM-1 and 3-epi-DON	Not cytotoxic; does not impair intestinal barrier function in human epithelial cell model	DOM-1/3-epi-DON forms only 2 H-bonds with ribosomal A-site (vs. 3 for DON); no MAPKinase activation; LAB primarily adsorbs DON rather than enzymatically transforming it	[194]
	Non-LAB bacterial transformation products; in vivo test (benchmark)	DOM-1 and 3-epi-DON	Not toxic for piglets (7-day, 3 mg/kg dietary exposure)	No intestinal, hepatic, or lymphoid histological lesions; no pro-inflammatory cytokine overexpression; benchmark for acceptable detoxification endpoint	[195]
FB1	*Lactobacillus* sp. from silage; biodegradation	HFB1 (as intermediate metabolite)	The toxicity of HFB1 was not assessed in the same LAB study. However, Grenier et al. [197] have reported.	LAB (*Lactobacillus* sp.) from silage produced HFB1 during FB1 degradation; HFB1 toxicity data require a separate benchmark study	[196]
	Enzymatic deesterification → HFB1 (benchmark; non-LAB esterase)	HFB1	No hepatotoxicity; minimal intestinal effects in piglets (2-week in vivo); does not inhibit ceramide synthase	Benchmark evidence: HFB1 only weakly alters the sphinganine/sphingosine ratio vs. FB1, which strongly disrupts sphingolipid metabolism	[197]

Note: The benchmark explains the involvement of non-LAB organisms or purified enzymes.

## Data Availability

No new data were created or analyzed in this study.

## Abbreviations

LAB	Lactic Acid Bacteria
AFB1	Aflatoxin B1
AFM1	Aflatoxin M1
OTA	Ochratoxin A
DON	Deoxynivalenol
ZEA	Zearalenone
FB1/FB2	Fumonisin B1/B2
PAT	Patulin
GRAS	Generally Recognized as Safe
QPS	Qualified Presumption of Safety
EFSA	European Food Safety Authority
FDA	Food and Drug Administration
EPS	Exopolysaccharides
PLA	Phenyllactic acid
HACCP	Hazard Analysis and Critical Control Points
MAPKinase	mitogen-activated protein kinase
